# Transcriptome Characterization of *Dendrolimus punctatus* and Expression Profiles at Different Developmental Stages

**DOI:** 10.1371/journal.pone.0161667

**Published:** 2016-08-25

**Authors:** Cong-Hui Yang, Peng-Cheng Yang, Jing Li, Fan Yang, Ai-Bing Zhang

**Affiliations:** 1 College of Life Sciences, Capital Normal University, Beijing, China; 2 Beijing Institutes of Life Science, Chinese Academy of Sciences, Beijing, China; Zhejiang University, CHINA

## Abstract

The pine moth *Dendrolimus punctatus* (Walker) is a common insect pest that confers serious damage to conifer forests in south of China. Extensive physiology and ecology studies on *D*. *punctatus* have been carried out, but the lack of genetic information has limited our understanding of the molecular mechanisms behind its development and resistance. Using RNA-seq approach, we characterized the transcriptome of this pine moth and investigated its developmental expression profiles during egg, larval, pupal, and adult stages. A total of 107.6 million raw reads were generated that were assembled into 70,664 unigenes. More than 30% unigenes were annotated by searching for homology in protein databases. To better understand the process of metamorphosis, we pairwise compared four developmental phases and obtained 17,624 differential expression genes. Functional enrichment analysis of differentially expressed genes showed positive correlation with specific physiological activities of each stage, and these results were confirmed by qRT-PCR experiments. This study provides a valuable genomic resource of *D*. *punctatus* covering all its developmental stages, and will promote future studies on biological processes at the molecular level.

## Introduction

The forest coverage of China is relatively low, and more than half of the forests consist of coniferous trees. The pine moth, *Dendrolimus punctatus* Walker (Lepidoptera: Lasiocampidae) is a serious pest of coniferous trees [1–6]. The caterpillar stage of this moth feeds on the leaves of conifers, especially masson pine (*Pinus massoniana*), and may cause their heavy and sometimes total defoliation, dieback, and death [3]. The moth species is native to south of China and could produce up to four generations per year [1, 2, 6]. It can potentially outbreak particularly in areas of low rainfall or during periods of drought [4]. In outbreak years, the rampant defoliators eat up a tree to death in a short time, causing withering and death of pine forest trees in large-scales. Furthermore, the caterpillars cover the roads and frequently cause human and livestock poisoning [3], causing significant ecological, economic, and social problems.

Field investigations found that type of diet affects the growth of pine moths. During development, the larvae have diverse feeding preferences to different stages of pine needles. Moreover, the damaged pine forests would influence the sexual ratio of pupae and the fecundity of adults [7–9]. To better explain these phenomena of pine moth, it is necessary to carry out extensive physiological studies and acquire sufficient genetic information. Nevertheless, the present molecular research have been merely focused on a few genes and their aspects, such as using COI sequences for species delimitation [10], phylogenetic studies using mitochondrial genomes [11], sex pheromone components [12], and odorant-binding proteins identification [13]. The present data is far from adequate for systematically elucidating molecular mechanisms underlying development and reproduction of *D*. *punctatus*.

The advent of next-generation sequencing technologies has profoundly improved traditional research and become one of the principal approaches for molecular analysis [14]. *De novo* transcriptome sequencing based on next-generation sequencing allows researchers to study organisms even without a reference genome and obtain gene expression profiles, novel transcripts, alternative splicing, and other important information [15]. With this rapid, sensitive, and accurate new method, multiple studies have been carried out that explore genetic mechanisms of adaptive evolution in insect morphology, physiology, behavior, and development [16–19].

In this study, we focused on an important forest pest, the pine moth *D*. *punctatus* (Walker), and performed the comprehensive transcriptome analysis during its four life stages–egg, larva, pupa, and adult. Through high-throughput sequencing technology, we generated more than one hundred million high quality raw reads, assembled approximately seventy thousand unigenes, of which about thirty percent matched known proteins. We also identified and characterized approximately twenty thousand differentially expressed genes during development process. These data provides a comprehensive transcriptome resource of *D*. *punctatus*, covering all the developmental stages, which can be further investigated for screening and analyzing of functional genes of interest.

## Materials and Methods

### Insect rearing

The pine moth *D*. *punctatus* sequenced in this study was originally obtained from Guilin in Guangxi Province, China. The larvae were reared in the laboratory climate chambers at 27±1°C, a photoperiod cycle of 16 h light: 10 h darkness and 70±5% relative humidity. Multiple replicates for each sample from different developmental stages were collected: fifty eggs, ten larvae each from 1^st^ to 3^rd^ instar, three larvae each from 4^th^ to 6^th^ instar, two pupae, and one male and one female adult. These samples were immediately frozen in liquid nitrogen and stored at—80°C for further use.

### RNA isolation and Illumina sequencing

Total RNA was extracted using the RNeasy Mini Kit (Qiagen, Germany) following the manufacturer’s protocol. RNA integrity was assessed using the RNA Nano 6000 Assay Kit of the Agilent Bioanalyzer 2100 system (Agilent Technologies). A mixture of total RNA from different developmental stages at equal ratio was used for Illumina sequencing of *D*. *punctatus* transcriptome. Sequencing libraries were generated using the NEBNext Ultra RNA Library Prep Kit (NEB, USA) according to the manufacturer’s protocol and sequenced on the Illumina Hiseq 2000 platform to obtain100 bp paired-end reads.

### *De novo* transcriptome assembly, gene annotation and classification

Before *de novo* assembly, the raw sequences were filtered to remove adaptor fragments, reads containing unknown nucleotide “N” over 5% and low quality reads with more than 20% of Q-value < 10 bases. The remaining clean reads were used for further analysis. Trinity pipeline [20] was used to assemble the transcriptome data, which consisted of three software modules: Inchworm, Chrysalis and Butterfly. Inchworm first assembled the read data set into a collection of linear contigs, and then Chrysalis clustered contigs into clusters and constructed complete de Bruijn graphs. Butterfly reconciled the graph with reads and pairs and ultimately reported full-length transcripts. To reduce the redundancy, the assemblies were clustered using the TGICL software [21] according to the pairwise sequence similarity, and the consensus sequences were produced for every cluster. The results were further filtered by cd-hit software [22], which clustered the sequences based on short word and selected the longest one as representative for every cluster. The raw data have been submitted to GenBank and deposited in the NCBI SRA under accession numbers SRX1330748.

To obtain the coding region and functional information, the filtered sequences were searched against NR, SWISSPROT, TrEMBL, and COG [23] protein databases using BLAST algorithm with an e-value cut-off of 1e-5. According to the aligned region of the best hit, the CDS and protein sequences were generated. Interproscan [24] was used to identify protein domains by searching against Interpro protein domain database [25]. To determine the distribution of gene functions at the macro level, gene ontology (GO) terms were assigned by Blast2GO [26] through a search of the NR database. The pathway analysis was performed using the KAAS webserver [27] from KEGG (Kyoto Encyclopedia of Genes and Genomes) database [28], which could identify significantly metabolic pathways or signal transduction pathways.

### DGE libraries sequencing and analysis

For comparative differential gene expression profiling during developmental stages of *D*. *punctatus*, four digital gene expression (DGE) libraries were constructed separately from eggs, larvae, pupae and adults. RNA was extracted as described above. The library products were then sequenced via Illumina HiSeq 2500 using single-end technology. The raw data are available at the NCBI SRA with accession numbers SRX1332929, SRX1332930, SRX1332932, and SRX1332933. Before mapping reads to reference database, the raw sequences were filtered to remove adaptor sequence and low quality sequences.

For annotation, clean reads were mapped to the reference sequences generated from the *D*. *punctatus* transcriptome using Tophat [29]. Gene expression levels were measured using the RPKM criteria [30]. To minimize the influence of differences in RNA output size between samples, the number of total reads were normalized by multiplying with normalization factors, as suggested by Robinson [31]. Differentially expressed genes were detected using the method described by Chen *et al* [16], which was constructed based on Poisson distribution [32] and eliminated the influence of RNA output size, sequencing depth and gene length. GO enrichment analysis for the differentially expressed genes was carried out based on an algorithm presented by GOstat [33], with the complete annotated gene set as the background. The *p*-value was approximated using the Chi-square test. Fisher’s exact test was used when any expected value of count was below 5. This program was implemented as a pipeline [16]. We used the Benjamini-Hochberg method to calculate false discovery rate (FDR, FDR < 0.05) for multiple testing [34]. Visualization of data was accomplished using the R packages VennDiagram (http://cran.r-project.org/web/packages/VennDiagram/index.html), pheatmap (https://cran.r-project.org/web/packages/pheatmap/index.html), and ggplot2 (www.ggplo2.org).

### Quantitative real-time PCR (qRT-PCR) validation

To validate the gene expression profiles post DGE data analysis, qRT-PCR was done using the Power SYBR Green PCR master mix (ABI, USA) and a CFX96 Real-Time PCR detection system (Bio-Rad, USA). Primer sequences of selected genes were designed by Primer5 program and listed in S9 Table. Total RNA was isolated as described for the DGE library preparation. The isolated RNA was purified by TURBO DNA-free DNase (Ambion, USA) and reverse-transcribed to cDNA using SuperScript III First-Strand Synthesis System (Invitrogen, USA). The qRT-PCR reactions were performed in three biological and technical replicates respectively, after which the average threshold cycle (Ct) was calculated per sample. *Actin* was used as endogenous control gene to normalize expression levels. The quantitative variation of genes expression was calculated using the 2^-ΔΔCT^ method [35].

## Results

### *De novo* sequencing and assembly of the *D*. *punctatus* transcriptome

**Fig 1:**
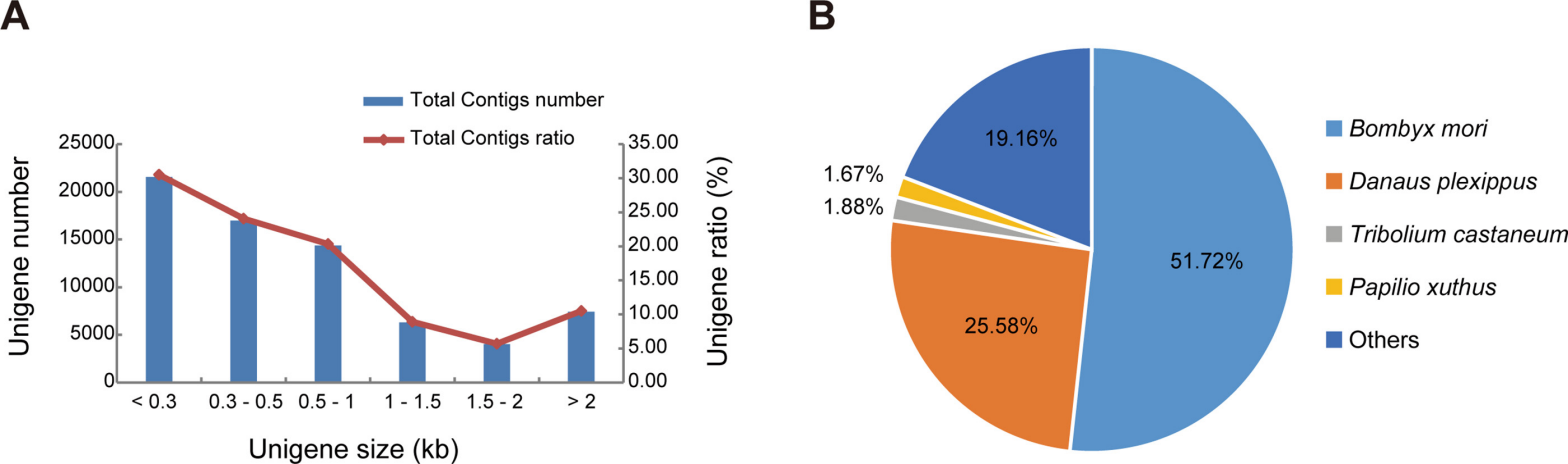
The statistics of assembly and homology search. (A) Length distribution of unigenes of *D*. *punctatus* transcriptome. (B) Species distribution of the BLASTX against Nr database, proportions of more than 1% was shown.

To comprehensively obtain the *D*. *punctatus* transcriptome, a library consisting of all its life stages including egg, larva, pupa, and adult, was constructed and sequenced, which produced 107. 6 million raw reads in total. After removing the low quality reads, we obtained 100,437,432 bp clean reads with 97.29% Q20 percentages (1% sequencing error rate) and 46.20% GC percentages, respectively (Table 1). These clean reads were *de novo* assembled into 83,596 contigs with an N50 of 1,902 bp using Trinity [20]. In order to reduce the redundancy of unigenes, we filtered the initial assembly using the TGICL package [21] and then clustered the results using the cd-hit package [22]. Finally, we obtained a transcript set of 70,664 unigene sequences with an N50 of 1,600 bp (Table 2). The size distribution of non-redundant unigenes is shown in Fig 1A and 26,618 sequences were longer than 1 kb. The length of unigenes ranged from 201 bp to 28,468 bp, with an average sequence length of 878 bp. There were about 15% unigenes filtered via the process of redundancy reduction, and rest of the sequences were used for annotation.

**Table 1:** Summary statistics of sequencing.

	Mix	Eggs	Larvae	Pupae	Adults
Raw data	107,634,026	13,257,859	11,173,753	11,752,032	11,690,370
Clean data	100,437,432	12,842,965	11,105,628	11,563,485	11,417,671
Q20%^†^	97.29%	98.81%	98.89%	98.91%	98.69%
GC%^‡^	46.20%	46.06%	45.91%	49.94%	48.02%

Note:

^†^The proportion of bases with quality value ≥ 20.

^‡^GC base content.

**Table 2:** Assembly statistics of *D*. *punctatus* transcriptome.

	Trinity	Trinity-TGICL	Trinity-TGICL-CDHIT
Total sequences	83,596	78,621	70,664
Total bases	85,595,998	78,884,595	62,090,047
Sequences length > 1kb	25,721	23,459	17,736
Average sequence length	1,023	1,003	878
N50 length	1,902	1,878	1,600

### Annotation of the predicted proteins

To get the coding region and functional information, a total of 21,444 unigenes were annotated by searching the protein database of NR, SWISSPROT, and TrEMBL using BLAST with an e-value of 1e-5. Because of the lack of reference genome and very little genetic information about *Dendrolimus* species, a relatively large proportion of unigenes (70% of all transcripts) could not be annotated accurately (S1 Table). There were about 16,754 unigenes that had best hits in NR database, and the distribution of species that matched are shown in Fig 1B. Of these, more than half the sequences (51.72%) were best matched with *Bombyx mori* sequences, followed by *Danaus plexippus* (25.58%), *Tribolium castaneum* (1.88%), and *Papilio xuthus* (1.67%).

### Unigenes functional classification and metabolic pathway analysis

**Fig 2:**
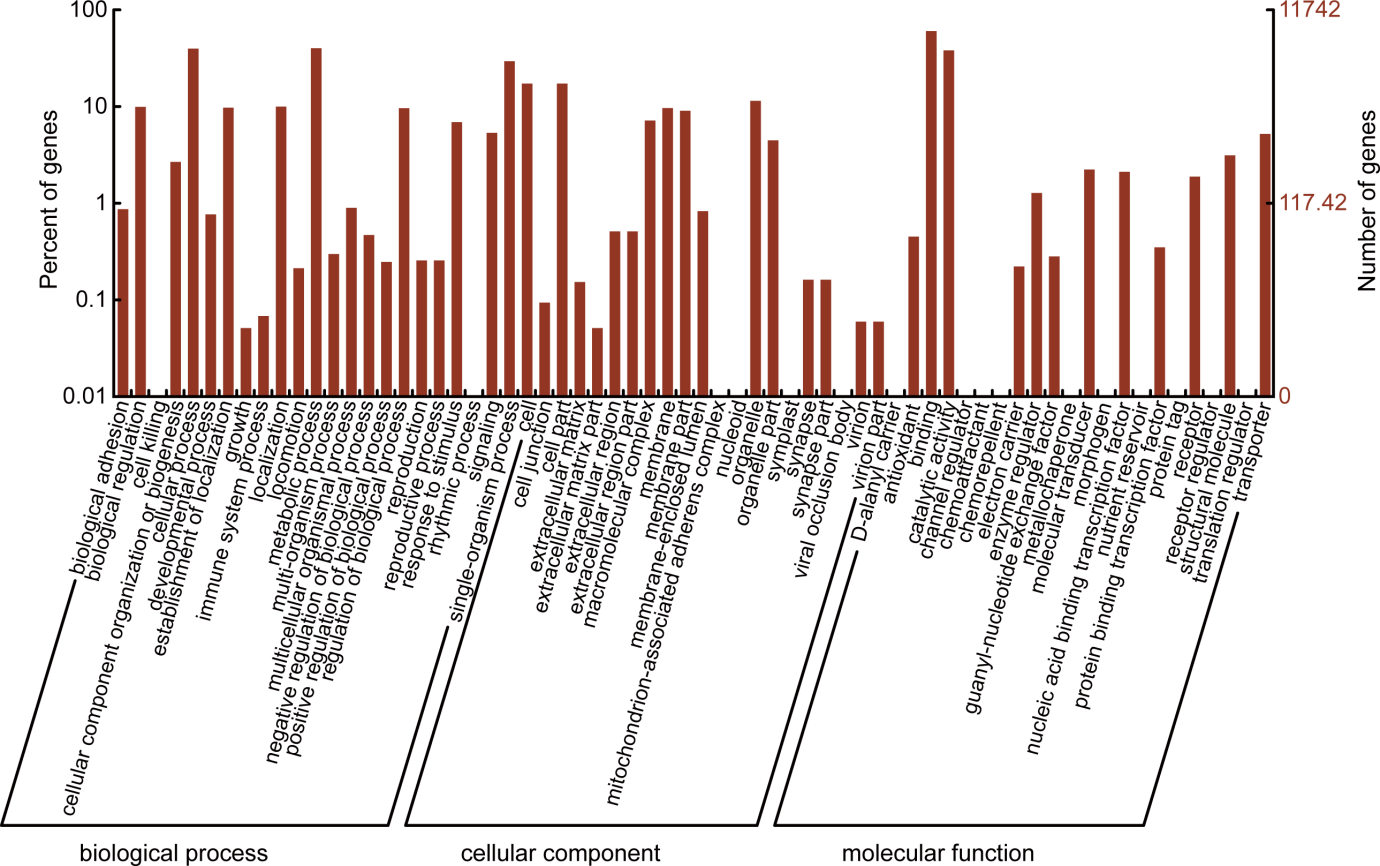
Histogram presentation of Gene Ontology classification. A total of 11742 unigenes were categorized into three main ontologies (Biological Process, Molecular Function, and Cellular Component) and 52 function groups. The right y-axis indicates the number of genes in the category. The left y-axis indicates the percentage of a specific category of genes in that main category.

Gene ontology (GO) analysis was used to predict the unigene functions at the macro level. After searching against Interpro database [25] and identifying protein domains, GO annotations were extracted from the IPR entry. 11,742 unigenes were annotated using 3,745 GO terms ranging from level 1 to level 11, which categorized them into three main ontologies and 52 function groups (Fig 2). The GO classification results indicated that the dominant categories were “metabolic process” (48.25%) and “cellular process” (45.17%) in the biological process ontology, “cell” (19.47%) and “cell part” (19.47%) in the cellular component ontology, and “binding” (68.92%) and “catalytic activity” (45.41%) in the molecular function ontology, respectively. The two categories of “metallochaperone activity” and “protein tag” were the smallest groups having only two and one unigenes, respectively.

**Fig 3:**
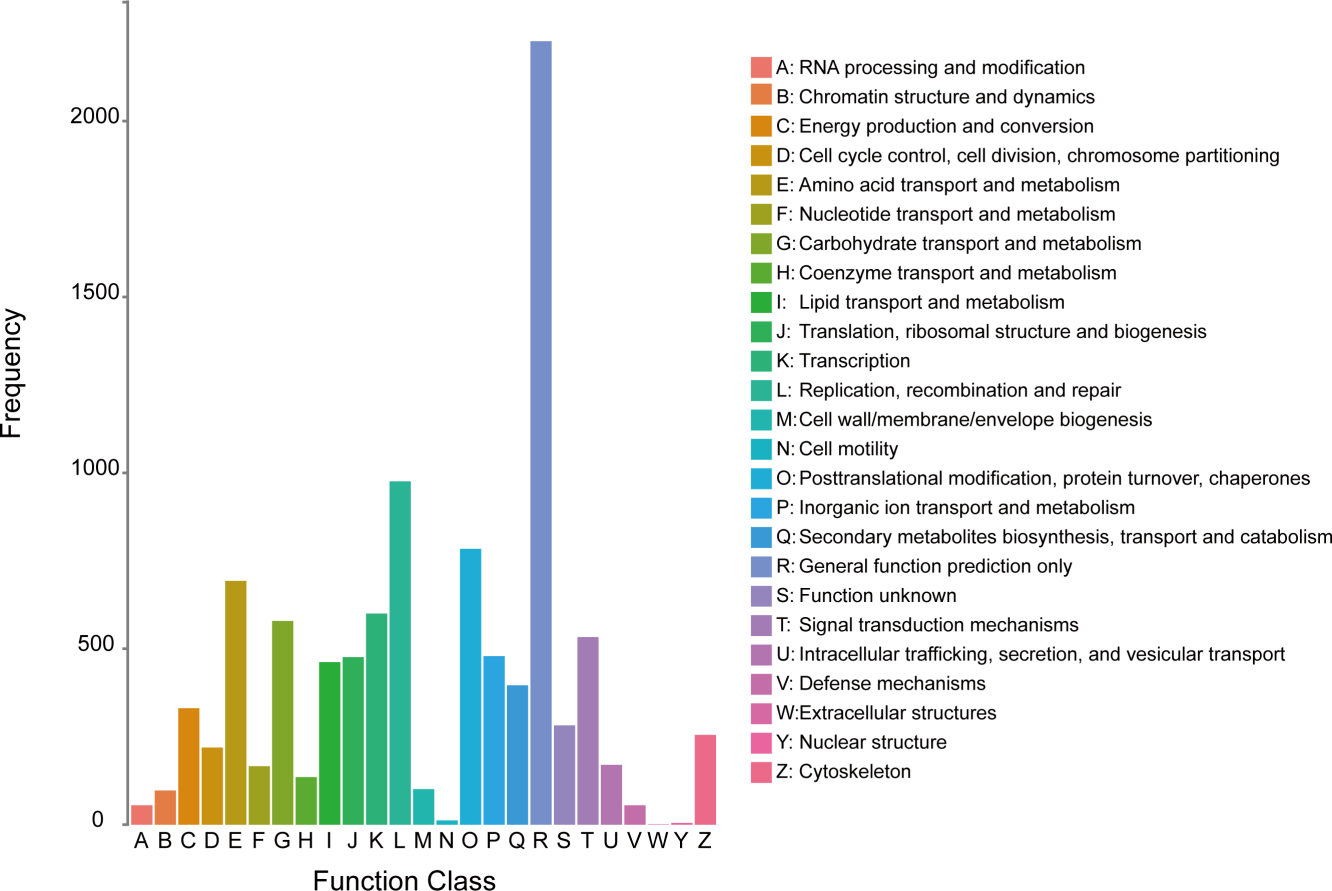
Classification of the clusters of orthologous groups (COG) for the transcriptome of *D*. *punctatus*. Based on sequence homology, a total of 10,089 unigenes were divided into 25 molecular families. The three most enriched clusters were “General function prediction only”, “Replication, recombination and repair”, and “Posttranslational modification, protein turnover, chaperones”.

We also annotated the unigenes by searching COG database to classify the functions of the predicted proteins. Based on sequence homologies, a total of 10,089 unigenes (47.05% of annotated transcripts) obtained COG annotations and were divided into 25 molecular families (Fig 3). The three most enriched clusters in COG analysis were “General function prediction only” (22.07%), “Replication, recombination and repair” (9.67%), and “Posttranslational modification, protein turnover, chaperones” (7.77%). “Cell motility” (0.12%), “Nuclear structure” (0.05%), and “Extracellular structures” (0.01%) were the smallest groups.

Metabolic pathway analysis was further performed on all of the unigenes using KEGG database [28]. In total, 5,272 unigenes were mapped to 306 KEGG pathways (S2 Table). The most representative pathways included Huntington's disease (4.22%), Ribosome (4.19%), Purine metabolism (4.13%), Spliceosome (4.00%), RNA transport (3.91%), and Protein processing in endoplasmic reticulum (3.91%). This annotation data provides a valuable molecular information resource for further understanding specific processes and functions in pine moth.

### Transcriptome profiling comparing different developmental stages of pine moth

**Fig 4:**
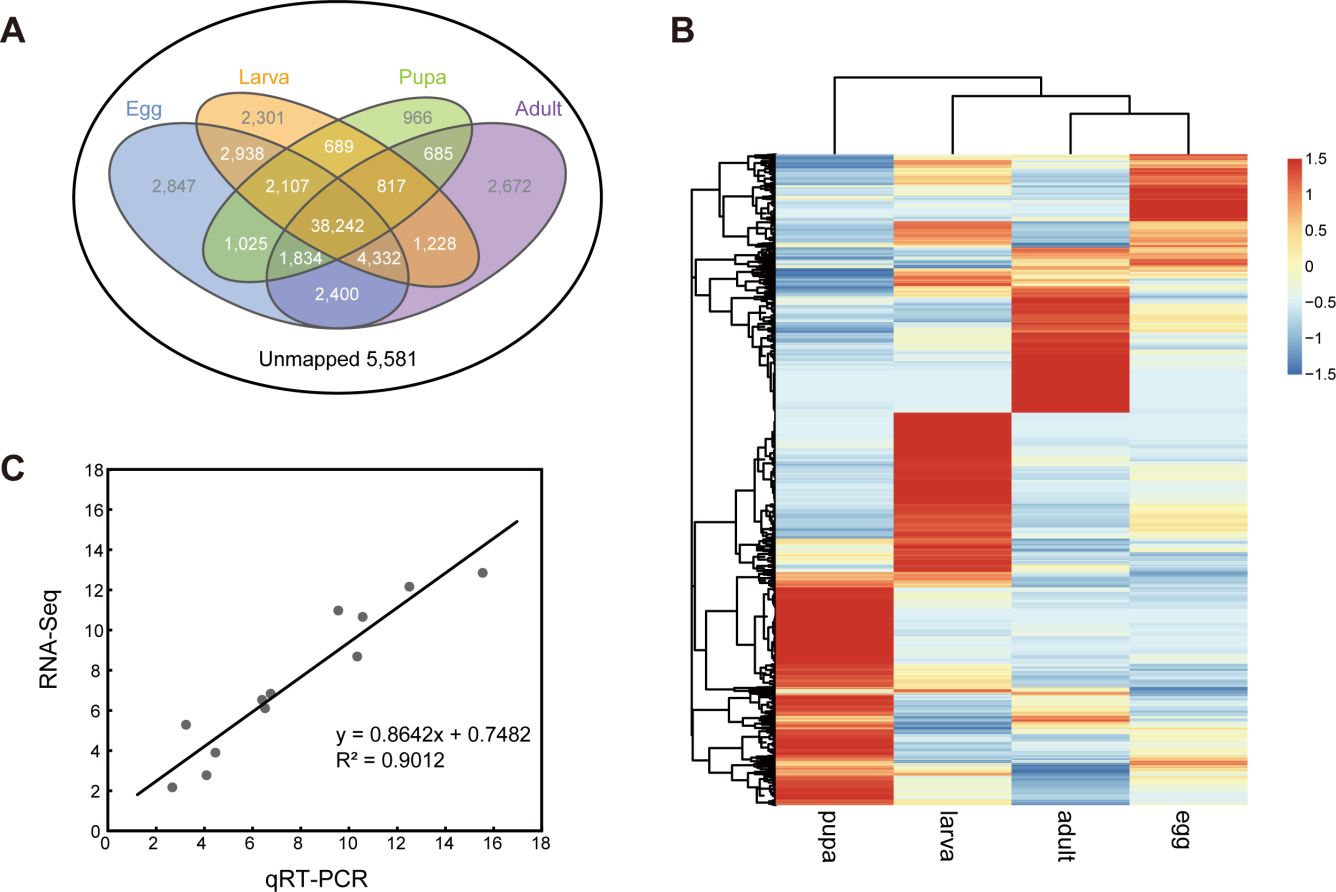
Comparison of transcriptome profiles of four developmental stages. (A) Venn diagram shown the unique and shared unigenes during *D*. *punctatus* development. (B) Heatmap of gene expression profiles using differently expressed genes in S4 Table. The color scales represent the log_2_-transformed RPKM values of four developmental stages. (C) qRT-PCR validation of DGE results.

Gene expression profiling is an important resource to understand the molecular mechanisms encompassing the different developmental stages of *D*. *punctatus*. We constructed four DGE libraries for the egg, larva, pupa, and adult and obtained approximately ten million raw reads in each library (Table 1). Post quality control, the available clean reads for each sample were mapped to the reference transcriptome database constructed above. Finally, amongst 70,664 reference transcripts, a total of 65,083 unique unigenes (92.10%) were detected in the four DGE libraries (S3 Table). The distribution of genes expression during development is shown in Fig 4A, with 2,847, 2,301, 966, and 2,672 genes specifically expressed in stages of egg, larva, pupa, and adult, respectively. In addition, there were 38,242 genes having expression in all of four stages, which accounts for 54.12% of the reference transcripts.

To measure the genetic landscape we evaluated the level of gene expression using RPKM method [30], as there were numerous common genes captured in different stages. We then performed three comparisons *viz*. egg *vs*. larva, larva *vs*. pupa and pupa *vs*. adult, and identified the genes showing significant variation in expression (FDR<10E-6, fold-change>1). A total of 11,914 genes were captured in the three comparisons (S4 Table), and then they were hierarchically clustered (Fig 4B). In the heat map, the gene expression profiles differed noticeably between the developmental stages that each comprised of a main cluster of high expression genes. For example, in the egg stage many crucial embryogenesis related genes were identified [36], comprising of maternal genes such as *nanos*, *vasa*, *mago nashi*, *squid*, *dorsal*, *easter*, and *snake*, gap genes such as *hunchback*, *kruppel*, and *caudal*, pair-rule genes such as *odd-paired*, *hairy*, and *even-skipped*, segment polarity genes such as *patched*, *engrailed*, and *gooseberry*, homeotic genes such as *proboscipedia*, *antennapedia*, and *ultrabithorax* (S7 Table). The previous studies had identified 79 embryonic developmental genes in *Bombyx mori* [37], and almost half of them (37 genes) were homologous with the pine moth genome. The other three stages also had their own striking high expression genes, such as *cuticle proteins* in larvae and pupae, and *chorion proteins* in adults (S3 and S4 Tables).

**Fig 5:**
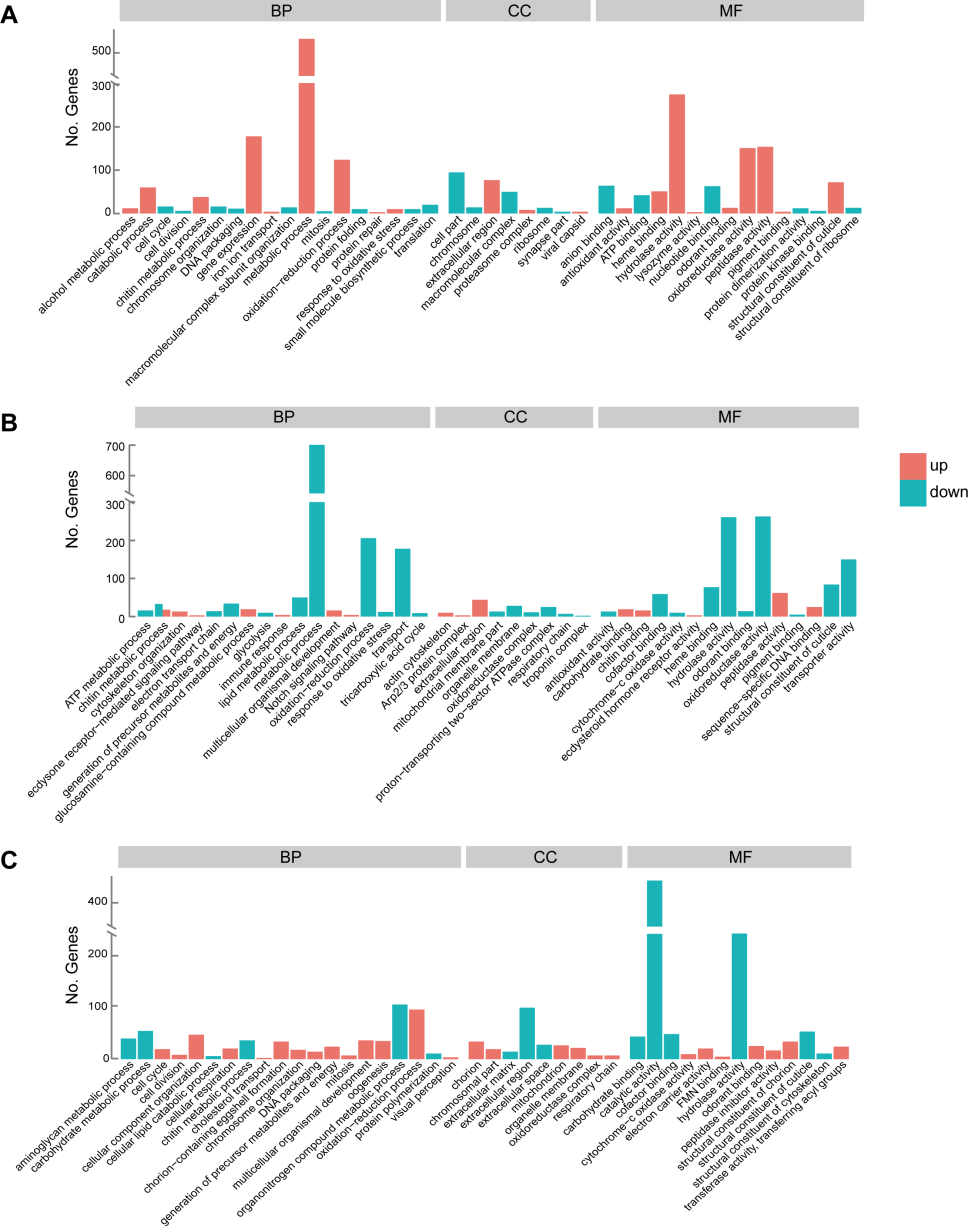
The representative GO terms enriched by differently expressed genes between developmental stages. Numbers of genes that were upregulated (red) or downregulated (blue) in comparisons of (A) egg versus larva, (B) larva versus pupa, and (C) pupa versus adult are shown. BP: biological process; CC: cellular component; MF: molecular function.

**Fig 6:**
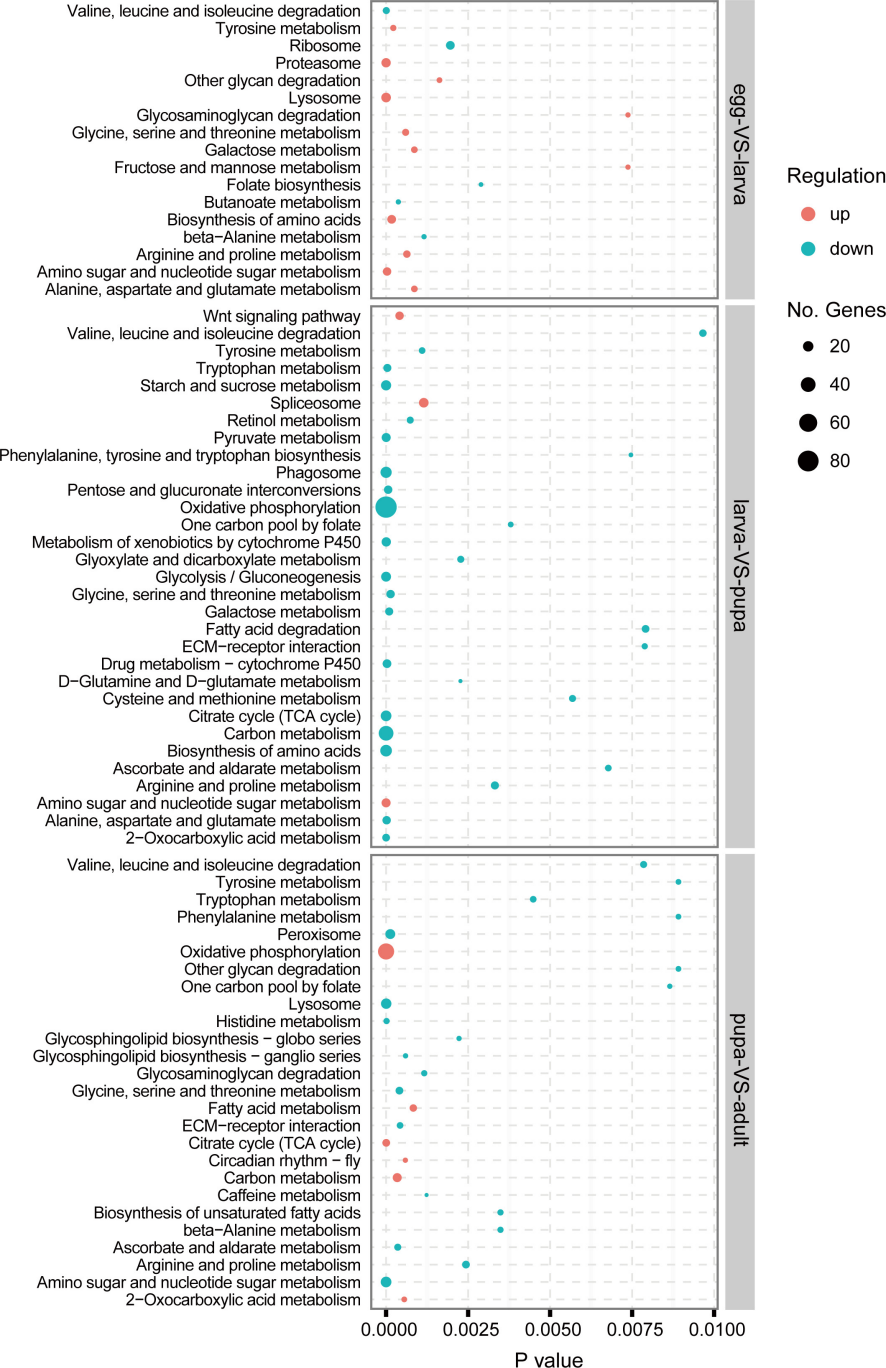
Enrichment analysis of KEGG pathways in comparisons of different stages. The x-axis indicates the p value calculated in enrichment test. The size of circles indicates the number of genes in that pathway. Red circles represented upregulated genes, while blue circles represented downregulated genes.

During the transformation from egg to larva, a total of 4,178 unigenes were identified that significantly changed their expression. Of these genes 2,919 were found to be up-regulated and 1,259 were down-regulated (S4 Table). The upregulated genes with the highest expression variation included *collagen*, *lipase*, *osiris*, *cuticular protein*, and *keratin-associated protein*. The down-regulated genes with the highest expression variation were *histone*, *lysozyme*, *homeobox protein ceh-43-like*, *cytochrome P450*, *endonuclease-reverse transcriptase*, *cyclin-1*, *glutamine synthetase*, and *fatty acid synthase*. According to the GO enrichment analysis (Fig 5A and S5 Table), most of the up-regulated genes in the larva were categorized into metabolic processes such as protein metabolic process, carbohydrate and carbohydrate derivative metabolic process, lipid metabolic process, and chitin metabolic process. Other categories, such as gene expression, oxidation-reduction process (biological process ontology), extracellular region (cellular component ontology), hydrolase activity, oxidoreductase activity, and heme binding (molecular function ontology), were also enriched. On the other hand, the high expressing genes in the egg stage were mainly enriched for cell division processes such as chromosome organization, cell cycle, DNA packaging, protein folding, and protein synthesis processes such as translation, ATP binding, ribosome. Furthermore we also performed KEGG analysis (Fig 6 and S6 Table). Lysosome, proteasome, and multiple metabolic pathways were up-regulated, and ribosome, folate biosynthesis, and valine, leucine and isoleucine degradation pathways were down-regulated.

Comparison between larval and pupal stage identified a total of 6,780 differentially expressed genes that included 2,691 up-regulated and 4,089 down-regulated genes (S4 Table). The most up-regulated genes included cellular metabolism related genes such as *D-amino-acid oxidase*, *UDP-glycosyltransferase*, and oogenesis related genes such as *fatty acid synthase*, *alpha-tocopherol transfer protein*, and *chorion*. The most down-regulated genes included *chemosensory protein*, *odorant binding protein*, *sensory protein*, and *cuticular protein*, and digestive and metabolic genes such as *cytochrome P450*, *peritrophin*, *insect intestinal mucin*, and *chymotrypsin*. Based on the GO classification (Fig 5B and S5 Table), the metamorphosis development related genes increased their expression in the pupal stage, as the enriched terms consisted of multicellular organismal development, notch signaling pathway, and ecdysteroid hormone receptor activity. For the down-regulated genes, the representative abundant enrichment GO terms included metabolic process, and transport (biological process ontology), proton-transporting two-sector ATPase complex, mitochondrial membrane part (cellular component ontology), and odorant binding, heme binding (molecular function ontology). In the KEGG analysis (Fig 6 and S6 Table), the three up-regulated pathways enriched were spliceosome, wnt signaling pathway, and amino sugar and nucleotide sugar metabolism. And the down-regulated genes were primarily involved in metabolic pathways.

Comparison between pupal and adult transcriptomes revealed 6,666 genes with significant differences in expression, including 3,016 and 3,650 genes up- and down-regulated respectively (S4 Table). The most prominently varying genes included up-regulated *chorion protein*, *egg-specific protein precursor*, *troponin C*, *peritrophin*, and *sensory protein*, and down-regulated *pupal cuticle protein*, *fatty acid synthase*, *15-hydroxyprostaglandin dehydrogenase*, *metalloprotease*, and *hemolymph proteinase*. When pupae metamorphosed into adults, most up-regulated genes were enriched for GO categories including oxidation-reduction process, cellular component organization, multicellular organismal development, oogenesis, and odorant binding (Fig 5C and S5 Table). In contrast, the down-regulated genes were mainly enriched for organonitrogen compound metabolic process, carbohydrate metabolic process, carbohydrate metabolic process, catalytic activity, hydrolase activity, structural constituent of cuticle, and structural constituent of cytoskeleton. According to KEGG analysis (Fig 6 and S6 Table), the up-regulated genes were enriched for pathways such as circadian rhythm–fly, citrate cycle (TCA cycle), and fatty acid metabolism. Simultaneously there were multiple metabolic pathways enriched for down-regulated expression.

### Gene expression validation using quantitative real-time PCR

We used quantitative real-time PCR (qRT-PCR) to confirm the quality of the RNA-seq data and the relative expression estimates. One of the most representative high expressing genes for each developmental stage was selected, namely *tyrosine hydroxylase* (G6771), *larval cuticle protein* (G16940), *pupal cuticle protein* (G8872), *vitellogenin* (G584), respectively, which are crucial in the development of pine moth. These genes were amplified by qRT-PCR methodology and validated. The gene expression values showed strong correlation with the expression patterns obtained from the RNAseq data (ρ = 0.91, Fig 4C).

## Discussion

The pine moth *D*. *punctatu* is one of the major pests destroying conifers in the south of China, and their periodic explosions cause huge losses to the forest and the economy. Beyond the physiology and ecology studies, the genome can also provide key information to further understand this species. High throughput sequencing has largely facilitated in revealing the functional role of the genome for non-model species. In this study, based on Illumina sequencing, we obtained 107.6 million raw reads and 70,664 unigenes, of these 21,444 unigenes were annotated by searching homologies in protein databases. We further compared the gene expression profiles between four developmental stages *viz*. egg, larva, pupa, and adult of the moth by constructing DGE libraries, which revealed 17,624 differential expression genes. These transcriptome sequences could be valuable references to the future genetic studies.

The development of multicellular organisms begins from a zygote. With a large number of genes expressing in an orderly manner according to time and space, the zygote develops through a series of cell divisions and differentiation program and eventually becomes an embryonic organism [38]. Research on embryonic development is significant in elucidating biological growth and evolution. Based on sequence similarity, many crucial genes and transcriptional factors participating in embryonic development of model organisms were found to have the homologues in pine moth genome (S7 Table). For instance, the *vasa* gene, originally identified in *Drosophila*, is essential for abdomen development in early embryogenesis [39], and has been identified as a marker gene of germ cells [40]. The *nanos* gene is a core component of germplasm and is involved in the formation of anterior-posterior body axis during embryogenesis [41]. One of maternal genes, *Mago nashi*, is required for germ cell determination and delineation of the longitudinal axis of the embryo [42]. In addition, highly expressed genes of the egg included genes related to embryogenesis signal transduction such as *easter*, *toll*, the genes regulated cell cycle such as *cyclin-dependent kinase1*, and the major yolk proteins such as vitellin, egg-specific protein precursor [43–46]. The high expression of genes related to embryonic development and cell mitosis revealed the ongoing drastic cellular proliferation and differentiation in the egg of pine moth.

The larva is the only feeding stage during development of pine moth. During the larval stage, we discovered that numerous digestive enzymes like trypsin, aminopeptidase, and carboxypeptidase, had a higher expression than in other stages (S4 Table). Simultaneously, genes involved in tricarboxylic acid cycle and glycolysis, belonging to energy metabolic processes, were also up-regulated. The active processes during larval metabolism including protein, carbohydrate, and lipid metabolic processes (Fig 5B and S5 Table) indicated that the larvae began large-scale accumulation of nutrition and energy for their rapid growth as well as for the future metamorphosis and reproductive process. In addition, genes participating in muscle growth [18] including *myosin-2*, *troponin-c*, *myo-inositol oxygenase*, and *pro-resilin-like*, genes associated with sensory [13] such as *odorant binding protein*, *chemosensory protein 2*, and *sensory protein*, genes pertaining to metabolic detoxification [19] including *cytochrome P450s*, *carboxylesterases*, and *glutathione S-transferases*, were expressed at significantly higher levels in the larva compared to egg (S4 Table). The increased activities of these genes were conducive to larvae surviving in complex environment.

Larvae undergo a critical phase of metamorphosis and then transform to adults during pupal stage. In this stage, many larval organs, such as silk glands, midguts, and fat bodies are degenerated gradually, and the wing disc and other primordium, which originally are under the larval cuticle, grow rapidly into adult organs [47, 48]. The GO term of proteolysis and KO term of lysosome were enriched in the up-regulated genes in pupae compared to larvae and adults (S5 and S6 Tables), which proved to play an important role in the self-digesting process of larval organs mediated by apoptosis and autophagy in the pupal stage [49–51]. Previous evidence suggesting that programmed autophagy was induced by ecdysone [52], corresponded with the GO enrichment analysis results that several genes associated to these factors are enhanced in pupae (S5 Table). To form the structure of adult insect, we also found several genes that were related to cellular proliferation and differentiation and critical for embryogenesis were reactivated in pupae (S5 and S6 Tables), such as the genes involved in notch signaling pathway and wnt signaling pathway [53, 54]. During pupation, lysozyme (G18356) was significantly up-regulated and was one of the top ten up-regulated genes except cuticle proteins, which demonstrated that pupae had an increased immune defense mechanism compared with larvae [55].

As the final stage of metamorphosis development, adults reach sexual maturity and the behaviors like courtship, mating, and oviposit emerge. There was a continued decline in metabolic activities of nutrition as well as the pupal stage (S5 and S6 Tables), as pine moths stop feeding during these two stages. On the other hand, the energy metabolic genes increased their expression levels in order to provide enough energy for adult movements. Numerous storage proteins and transport proteins were expressed at a high level such as apolipophorin III (G13232), which displayed the highest expression value in adult DGE library (S3 Table) and plays an important role during insect flight and gametogenesis [56]. Some genes involved in multicellular organismal processes and reproduction were enhanced significantly in the adult stage, for instance, *vitellogenin* (G584, etc.), *chorion* (G36030, etc.), and *egg-specific-protein* (G9850) during oogenesis [46, 57], and *cellular retinoic acid binding protein* (G24698), *14-3-3 epsilon protein* (G7063), and *testis specific tektin* (G28833) during spermatogenesis [58]. In addition, some of high expressing genes in the adult were identified as olfactory genes (S8 Table) [13] comprising of *odorant binding proteins*, *chemosensory proteins*, *sensory neuron membrane proteins*, *odorant receptors*, *ionotropic receptors*, *etc*., which are essential to detect odorants in the environment for their survival and reproduction.

The surface of insect body is covered with cuticle. Insect cuticle cannot only protect against the attack of pathogens and survive in unfavorable environment, but also is required for the development and metamorphosis in life history [59, 60]. The main component of insect cuticle is chitin and cuticular proteins [61]. It was obvious that many cuticular proteins were identified as the most differentially expressed genes in the pairwise comparisons of each stage. In accordance with previous evidence, more than 1% of the protein-coding genes were cuticular proteins in the insect genome that have been sequenced [62]. Therefore cuticular protein is one of the major structural proteins of insects, and their further investigation would be meaningful for interpreting the molecular mechanism of insect molting in development.

In this study, we analyzed the transcriptome of the pine moth *D*. *punctatus* and described their expression profiles during development. By comparing four developmental stages, we found an obvious correlation between differentially expressed genes and specific physiological activities in each stage. In eggs, the genes involved in embryonic development and cell mitosis were up regulated. In larvae, the metabolism related genes and digestive enzymes had a higher expression. In pupae, the genes related to cell proliferation and differentiation reactivated, and the highly expressed genes in adults were significantly associated with the processes such as reproduction and flight. Many genes were also identified by homology in pine moth genome, which were identified to be necessary for significant biological processes of model organisms. Our results provide vast genetic information and will be a valuable reference for the further genetic researchers studying pine moth growth and development.

## Supporting Information

S1 Table Unigenes annotation by sequence similarity against the multiple protein databases. (XLSX)

S2 Table The KEGG pathway analysis of *D*. *punctatus* Transcriptome. (XLSX)

S3 Table The unigenes mapped in each DGE library of developmental stage. (XLSX)

S4 Table The differentially expressed genes between four different stages. (XLSX)

S5 Table GO enrichment analysis in comparisons of different stages. (XLSX)

S6 Table KEGG enrichment analysis in comparisons of different stages. (XLSX)

S7 Table Sequence information of unigenes related to embryonic development. (XLSX)

S8 Table The olfactory genes identified in *D*. *punctatus* Transcriptome. (XLSX)

S9 Table Primers used in qRT-PCR for confirmation of differentially expressed genes. (XLSX)
